# Supplementary material for: Generation and deposition of Aβ43 by the virtually inactive presenilin‐1 L435F mutant contradicts the presenilin loss‐of‐function hypothesis of Alzheimer's disease
Source: EMBO Mol Med. 2016 Mar 17;8(5):458–65. doi: 10.15252/emmm.201505952 (PMC5119496; doi:10.15252/emmm.201505952)
Supplement: Supplementary file 1 — Expanded View Figures PDF [file EMMM-8-458-s001.pdf]

Expanded View Figures

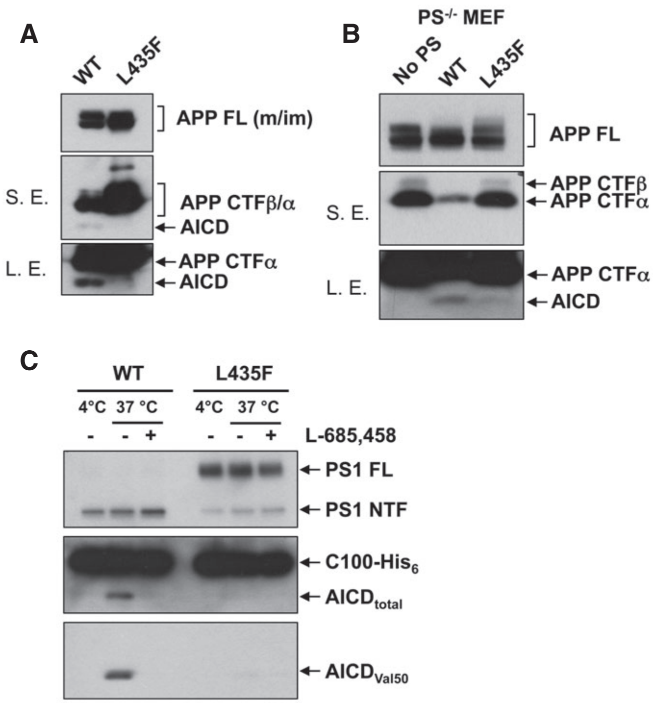

Figure EV1. The PS1 L435F FAD mutant strongly impairs AICD generation.

A Levels of full-length APP, APP CTFs, and AICD were analyzed in cell lysates of single-cell clones of HEK293/sw cells stably expressing PS1 WT or PS1 L435F by immunoblotting using antibody Y188. S.E., short exposure; L.E., long exposure.

B Levels of endogenous full-length APP, APP CTFs, and AICD were analyzed in cell lysates of PS1/2$^{-/-}$ MEF cells stably transduced with PS1 WT or PS1 L435F by immunoblotting using antibody Y188 as in (A). S.E., short exposure; L.E., long exposure.

C Cell-free generation of AICD from recombinant APP C100-His$_6$ substrate by CHAPSO-solubilized γ-secretase of stably transduced PS1/2$^{-/-}$ MEF cells was analyzed by immunoblotting using Penta-His (AICD total) and anti-AICD neo-epitope (AICD Val50) antibodies, respectively.

Source data are available online for this figure.

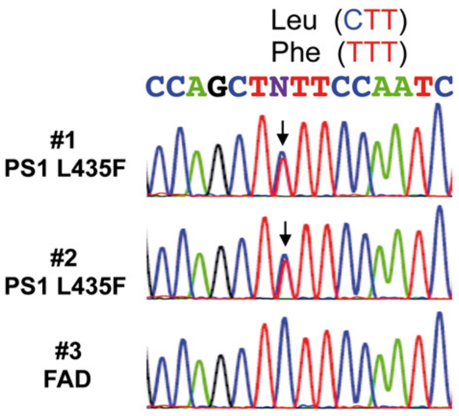

Figure EV2. DNA sequence analysis of PS1 exon 12.
A heterozygous C to T missense mutation at residue 435 changing leucine to phenylalanine is confirmed in two FAD cases (#1 and #2), but not found in a third control FAD case (#3).

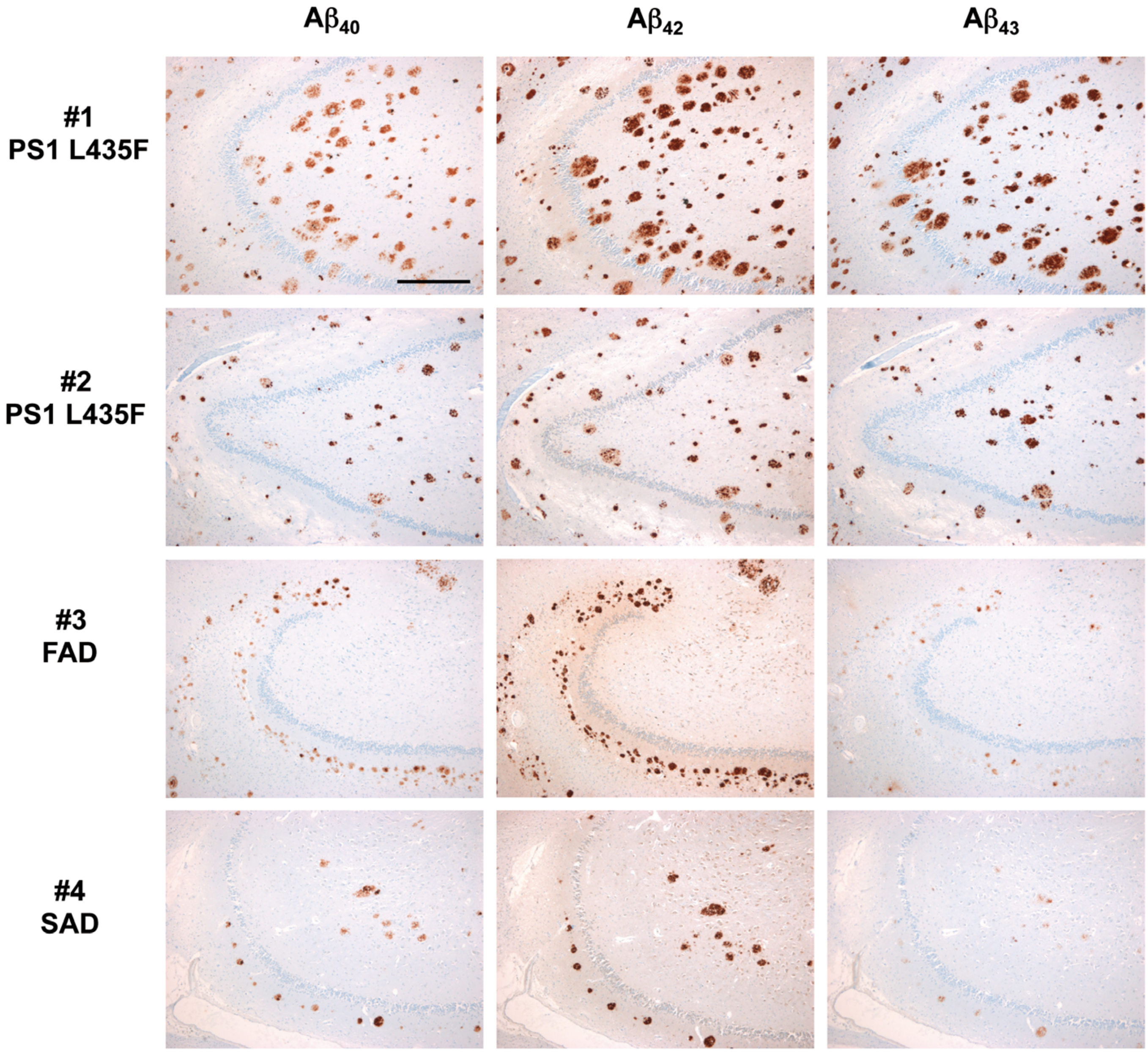

Figure EV3. Deposition of Aβ isoforms in the hippocampus of AD cases with and without PS1 L435F mutation.

Immunohistochemical detection of Aβ40 (left column), Aβ42 (medium column), and Aβ43 (right column) in consecutive hippocampus paraffin sections (hilus region) of two FAD cases with PS1 L435F mutation (cases #1 and #2, 1st and 2nd rows), one FAD case with different PS1 mutation (case #3, 3rd row) and one sporadic SAD case (case #4, fourth row). Identical to findings in the frontal cortex (Fig 3), plaques in both PS1 L435F cases contain abundant Aβ43 in contrast to plaques of cases without that mutation (cases #3 and #4). Aβ42 levels are similar in plaques of all cases, whereas Aβ40 levels seem to be slightly higher in plaques of PS1 L435F cases. Note that the plaque size in PS1 L435F cases (in particular in case #1) is much larger than in cases #3 and #4; these large plaques represent cotton wool plaques. Scale bar = 500 μm. Magnification is identical in all pictures.

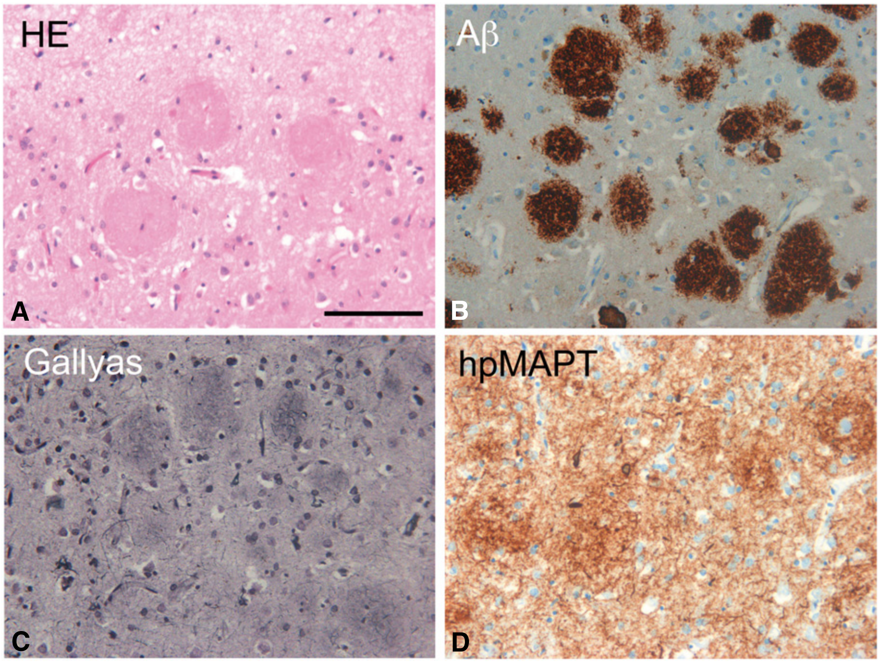

Figure EV4. Cotton wool plaques in the frontal cortex of PS1 L435F mutation case #1.

A In conventional hematoxylin–eosin (HE) stains, cotton wool plaques impress as round pink-colored structures in the neuropil. Scale bar = 100 μm. Magnification is identical in all pictures.

B Immunohistochemistry for Aβ (4G8 antibody) even visualizes plaques that are not visible in HE stains. Note that Aβ in cotton wool plaques is densely packed and that cotton wool plaques are large with diameters between 50 and 100 μm.

C A Gallyas silver stain identifies most cotton wool plaques as neuritic plaques. However, the number of dystrophic neurites (black threads) crossing these plaques is low.

D In immunohistochemical stains for hyperphosphorylated microtubuli-associated protein tau (hp-MAPT) (AT-8 antibody), a dense network of neuropil threads is seen. Agglomerations of threads represent neuritic cotton wool plaques.
