# Supplementary material for: Generation and deposition of Aβ43 by the virtually inactive presenilin‐1 L435F mutant contradicts the presenilin loss‐of‐function hypothesis of Alzheimer's disease
Source: EMBO Mol Med. 2016 Mar 17;8(5):458–65. doi: 10.15252/emmm.201505952 (PMC5119496; doi:10.15252/emmm.201505952)
Supplement: Supplementary file 2 — Table EV1 [file EMMM-8-458-s002.docx]

**Table EV 1 Autopsy cases**

F female, FAD familial Alzheimer disease, M male, PMI post mortem interval, SAD sporadic Alzheimer disease

| **Case** | **Age** | **Sex** | **PMI** | **Diagnosis** |
| --- | --- | --- | --- | --- |
| #1 | 59y | F | 20 hrs | FAD  (sibling of #2) |
| #2 | 55y | M | 24 hrs | FAD  (sibling of #1) |
| #3 | 51y | M | 8 hrs | FAD |
| #4 | 73y | M | 28 hrs | SAD |
