# Supplementary figures and images for: Generation and deposition of Aβ43 by the virtually inactive presenilin‐1 L435F mutant contradicts the presenilin loss‐of‐function hypothesis of Alzheimer's disease
Source: EMBO Mol Med. 2016 Mar 17;8(5):458–65. doi: 10.15252/emmm.201505952 (PMC5119496; doi:10.15252/emmm.201505952)

**A**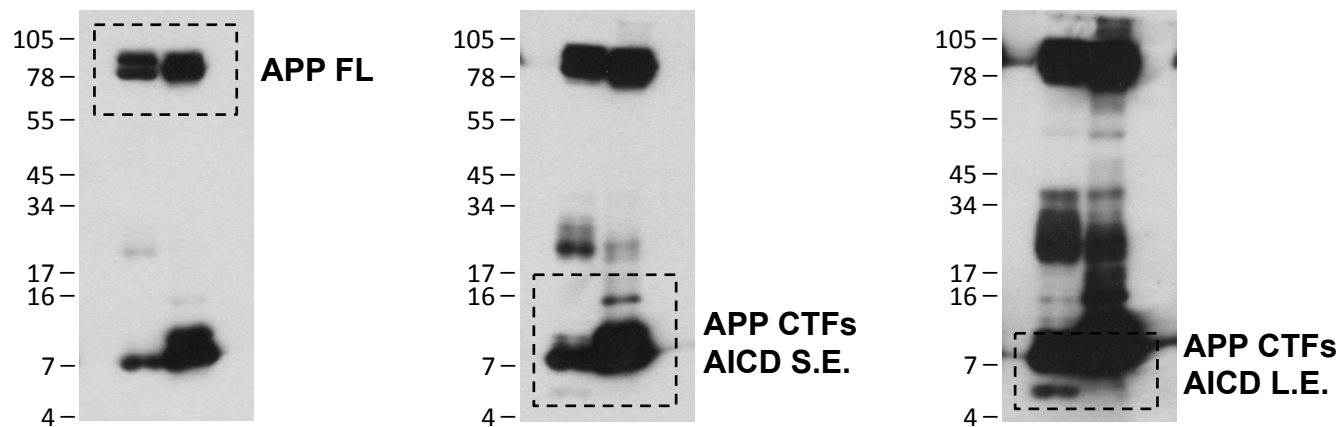**B**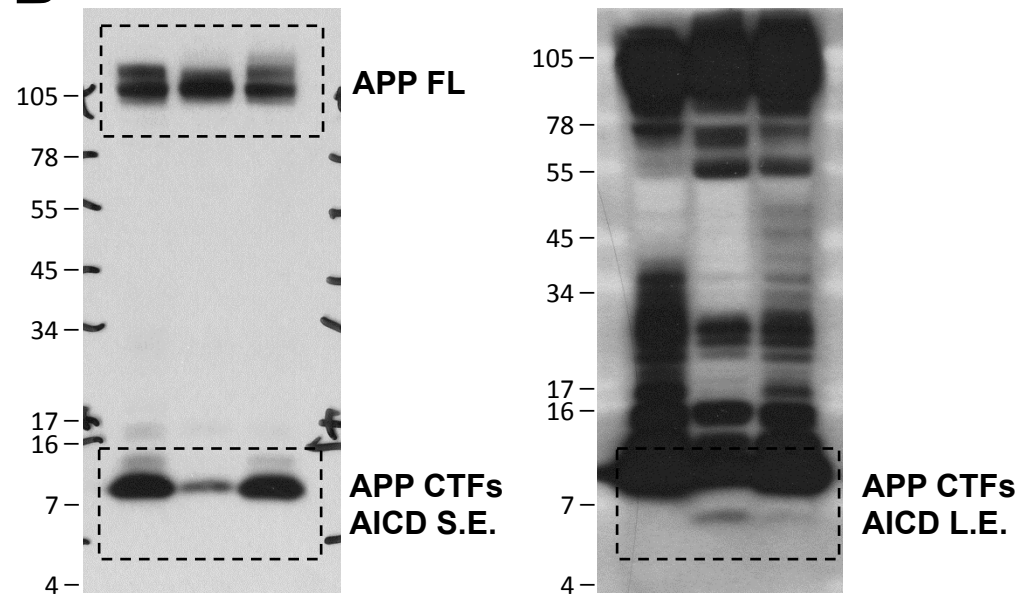**C**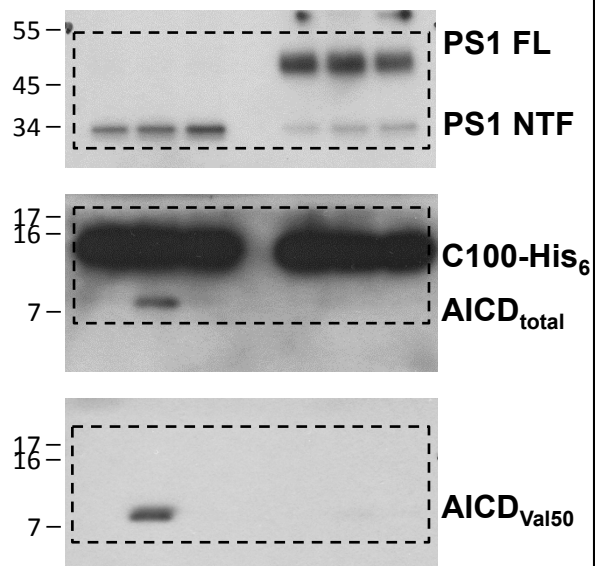

Supplement: Supplementary file 3 — Source Data for Figure EV1 [file EMMM-8-458-s005.pdf]

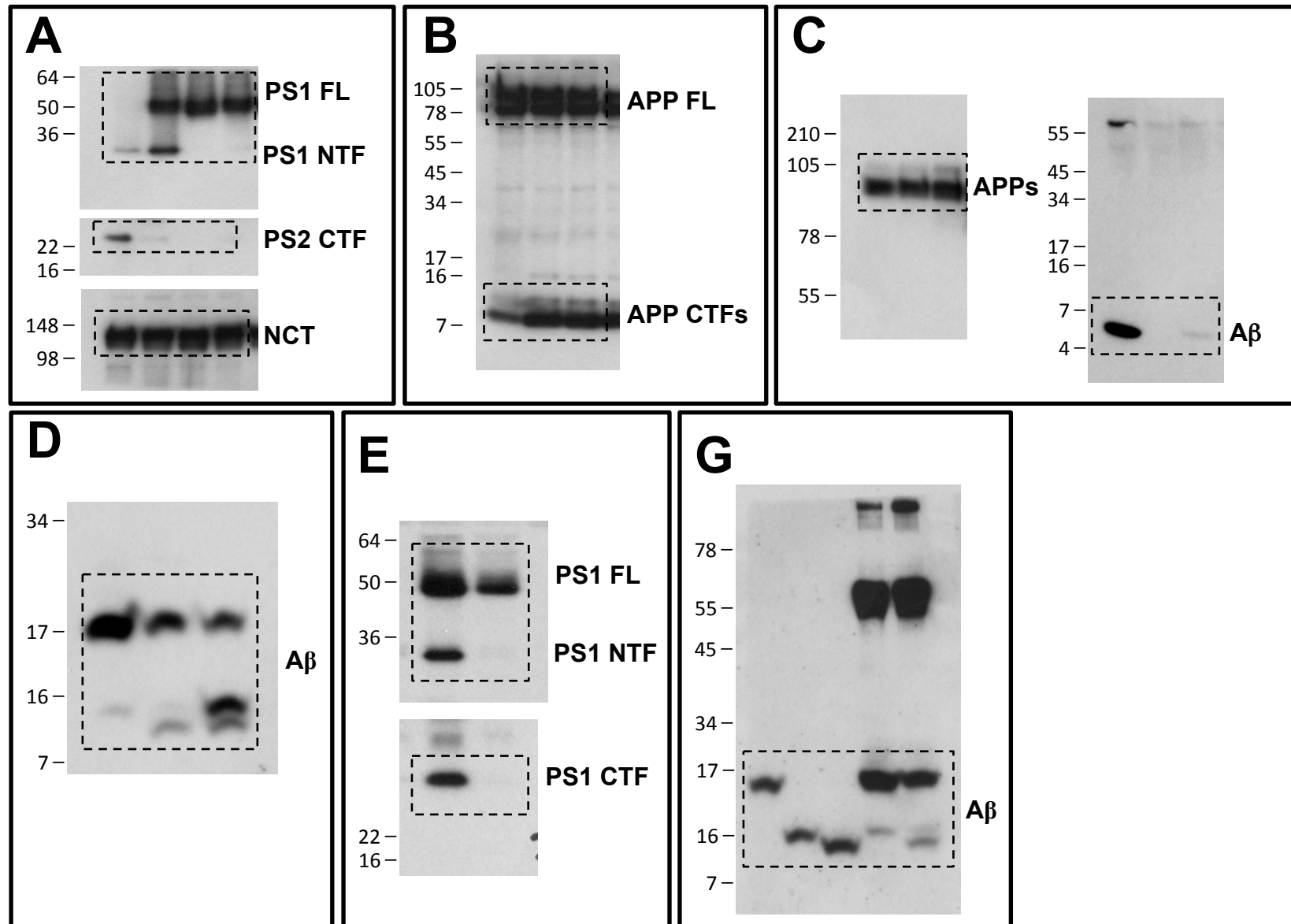

Supplement: Supplementary file 5 — Source Data for Figure 1 [file EMMM-8-458-s003.pdf]

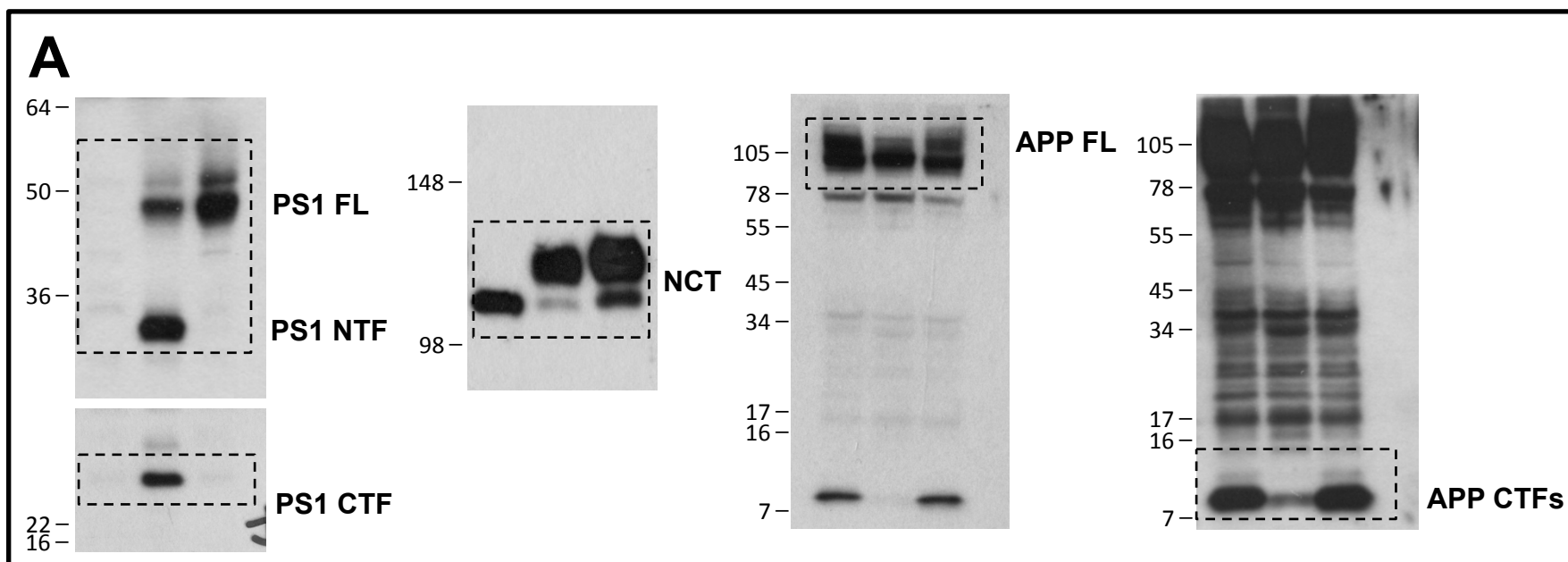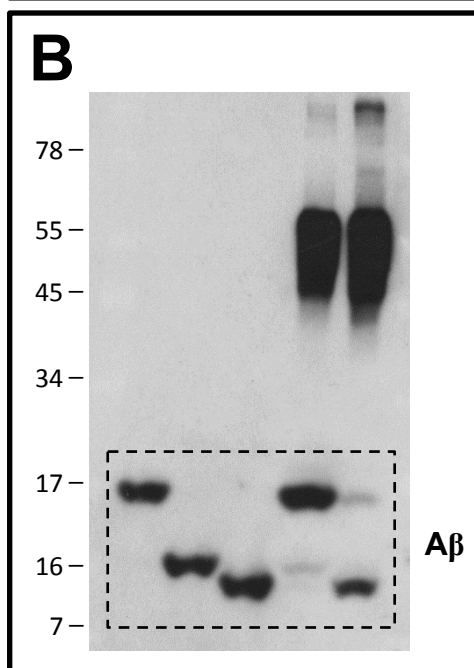

Supplement: Supplementary file 6 — Source Data for Figure 2 [file EMMM-8-458-s004.pdf]
